# Samba: A Real-Time Motion Capture System Using Wireless Camera Sensor Networks

**DOI:** 10.3390/s140305516

**Published:** 2014-03-20

**Authors:** Hyeongseok Oh, Geonho Cha, Songhwai Oh

**Affiliations:** Department of Electrical and Computer Engineering and ASRI, Seoul National University, 1 Gwanak-ro, Gwanak-gu, Seoul 151-744, Korea; E-Mails: hyeongseok.oh@cpslab.snu.ac.kr (H.O.); geonho.cha@cpslab.snu.ac.kr (G.C.)

**Keywords:** wireless camera sensor networks, real-time system, motion capture

## Abstract

There is a growing interest in 3D content following the recent developments in 3D movies, 3D TVs and 3D smartphones. However, 3D content creation is still dominated by professionals, due to the high cost of 3D motion capture instruments. The availability of a low-cost motion capture system will promote 3D content generation by general users and accelerate the growth of the 3D market. In this paper, we describe the design and implementation of a real-time motion capture system based on a portable low-cost wireless camera sensor network. The proposed system performs motion capture based on the data-driven 3D human pose reconstruction method to reduce the computation time and to improve the 3D reconstruction accuracy. The system can reconstruct accurate 3D full-body poses at 16 frames per second using only eight markers on the subject's body. The performance of the motion capture system is evaluated extensively in experiments.

## 1. Introduction

We are witnessing rapidly-growing 3D industries, such as 3D movies, 3D TVs and 3D smartphones. It is expected that the consumption of 3D content will be further increased as more 3D products are introduced into the market. While there are many ways to consume 3D content, 3D content generation is still an expensive task and remains in the hands of professionals. As we have seen from the analog to digital camera conversion in early 2000, an inexpensive way to create photos and videos has revolutionized the industry. As common users can create multimedia content with inexpensive cameras, the demand for digital cameras has become greater, further lowering the prices of hardware for content generation and consumption. In addition, the introduction of digital cameras has also revolutionized the Internet, as the creation and sharing of photos and videos have been accelerated with help from social network sites, such as YouTube, Flickr and Facebook. Based on these facts, it can be concluded that the success of the 3D market depends on the availability of an inexpensive tool, with which a common user can easily create 3D content. The present paper proposes a low-cost approach to 3D content generation.

Motion capture systems, such as the Vicon MX motion capture system [1], can provide the full 3D pose of a subject using a set of markers. They are commonly used in the film and gaming industry for creating realistic and complex 3D motions. While highly accurate 3D motions can be generated, motion capture systems are still too expensive for common users. For example, a typical entry-level motion capture system set with a minimum of three cameras costs more than $ 12,000.

**Figure 1.**
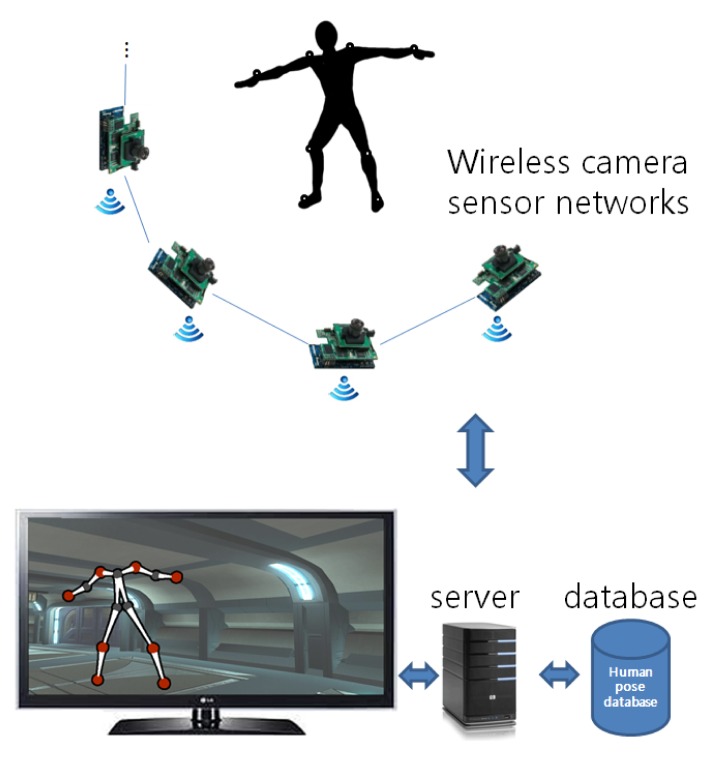
An overview of the Samba motion capture system.

A wireless camera sensor network consists of inexpensive camera nodes with wireless connectivity and has been applied to a number of applications, such as surveillance, environmental monitoring, traffic modeling, human behavior understanding, human pose estimation, assisted living and sports (see [2] and the references therein). In this paper, we design and implement a motion capture system using a wireless camera sensor network. An overview of the proposed system is shown in Figure 1.

The proposed motion capture system is named Samba. The Samba motion capture system consists of a wireless camera sensor network, a server and an optional display. Note that a server can be a notebook computer or a smart device, as in [2], to make the overall system more portable. A human subject wears markers on her joints and performs a series of motions in front of a wireless camera sensor network. Each camera node in a wireless camera sensor network detects markers and wirelessly transmits marker locations to the Samba server. The server then reconstructs the 3D motion using the received marker positions and a human pose database. By using a wireless camera sensor network, we have developed a portable personal human motion capture system. A user can easily carry and deploy camera nodes to collect 3D content. Furthermore, a minimal number of markers are used to make motion capture more convenient for common users. The Samba motion capture system can track moving marker sets and display 3D motion in real time. Currently, the system runs at 16 frames per second.

Samba is based on the data-driven 3D human pose reconstruction method, which utilizes a human pose database to reliably reconstruct human poses from noisy and missing observations from camera nodes. A human pose is reconstructed using the local pose space, which is consistent with the current observation. We propose pseudo-poses to better represent the local pose space with a dense set of data points. In experiments, the reconstruction error is reduced by 14% with a reduced computation time when pseudo-poses are used to represent the local pose space.

A newly introduced Kinect depth camera from Microsoft is a low-cost motion capture system. Kinect uses an infrared (IR) projector and cameras to construct a depth image. However, it is known that the Kinect camera has the following limitations: certain materials, such as hair and shiny surfaces, do not reflect infrared light, resulting in ‘drop out’ pixels; the ambient infrared can swamp the active signal, preventing any depth inference under bright sunlight; and the depth range is limited by the power of the emitter (the typical operating range of Kinect is about 4 *m*) [3]. A wireless camera sensor network-based motion capture system can operate under bright lighting conditions and is highly portable, *i.e.*, no wiring is required and a rapid deployment is possible. Hence, wireless camera sensor network-based motion capture systems have application domains that are different from Kinect-based systems.

The remainder of this paper is organized as follows. Related work in 3D motion capture is discussed in Section 2. An overview of the proposed Samba motion capture system is described in Section 3. In Section 4, the data-driven 3D human pose estimation method is presented. The system implementation of Samba is described in Section 5. The experimental results of the Samba motion capture system are presented in Section 6.

## 2. Related Work

A motion capture system is used for a wide range of applications, including sports, medicine, advertising, law enforcement, human-robot interaction, manufacturing, surveillance and entertainment [4,5]. A number of different methods have been developed for capturing human motions.

Wei and Chai reconstructed 3D human poses from uncalibrated monocular images in [6]. They assumed that all the positions of joints from images were known, and the camera was placed far from the human subject. These assumptions are based on the reconstruction method for an articulated object by Taylor [7]. In [7], the author formulated the 3D human pose reconstruction problem as an optimization problem using three sets of constraints: bone projection, bone symmetry and rigid body constraints. Magnus Burenius *et al.* [8] developed a new bundle adjustment method for 3D human pose estimation using multiple cameras. Their method is similar to [6], but temporal smoothness constraints are added and spline regression is used to impose weak prior assumptions on human motion. All three methods require known positions of joints and lengths between pairs of joints. Since it is not possible to reliably detect all markers autonomously from images due to self-occlusion, they are not applicable for a practical motion capture system.

Multiple cameras have been used to reconstruct 3D human poses using markers. In [9], four colored markers are used for extracting joints from two cameras. The locations of other joints are estimated using four marker positions and a silhouette of the subject. While it provides a low-cost solution, it cannot be run in real time, and the reconstruction error is too large to be used in practice. In [10], an optical motion capture system with pan-tilt cameras is proposed. The proposed motion capture system runs in real time and labels markers automatically. However, the system requires a set of pan-tilt cameras and computers. While the system is cheaper than commercial optical motion capture systems, it is still too expensive for common users.

The depth information can be used for 3D human pose estimation [3,11–13]. In [11], the authors developed a nonlinear optimization method based on the relationship between joint angles and an observed pose from a depth image for human motion estimation. In [12], notable image features, such as a head and a torso, are extracted from depth and color images, and 3D human poses are estimated using constrained optimization based on constraints among extracted image features. Baak *et al.* [13] presented a tracking framework for estimating 3D human poses from depth-image sequences by utilizing a pose database. Shotton *et al.* [3] proposed a method to estimate a 3D human pose from a single depth image by detection body parts using a randomized decision forest and estimating 3D joint positions from detected parts and depth information.

In [14], a 3D human pose reconstruction system using a wireless camera network was presented. The method detected five-upper body parts (a head, two shoulders and two wrists) and reconstructed an upper body in 3D in a heuristic manner. While our proposed system is similar to [14], our system is based on a more systematic approach based on multiview geometry and motion capture databases for improving accuracy at low computational cost. In addition, a full body reconstruction is possible in Samba.

Chai and Hodgins [15] have demonstrated that the use of a local, lower dimensional representation of a human pose gives a better pose estimation by eliminating ambiguity. The local pose space can be constructed using neighboring poses in the human pose database. In addition, the computation time required for the optimization routine can be reduced, since lower dimensional representations of human poses are used. The proposed system is designed based on the method suggested by [15] in order to reduce the computation time and to improve the 3D reconstruction accuracy, such that motion capture can be performed in real-time using a low-cost wireless camera sensor network.

## 3. An Overview of the Samba Motion Capture System

The Samba motion capture system is based on a wireless camera sensor network (see Figure 1). A wireless camera sensor network consists of a number of camera nodes (e.g., a CITRIC camera mote [16]). Each camera node has a camera, an on-board processor for image processing and wireless connectivity. We assume that a wireless camera sensor network has been deployed and that cameras are calibrated in advance, as described in [2].

**Figure 2.**
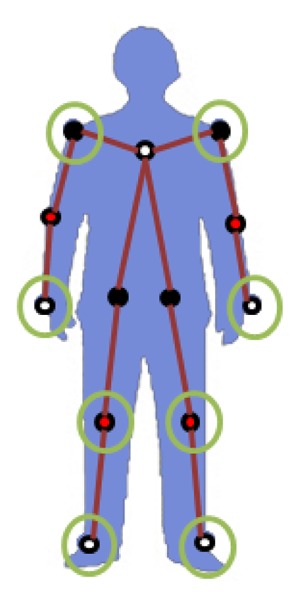
A 13-joint human skeleton model. The eight markers used by the Samba motion capture system are indicated with larger circles around the joint. Red colored joints have one degree of freedom, and black colored joints have two degrees of freedom.

The goal of the Samba motion capture system is to reconstruct 3D poses of a human subject based on images taken from multiple camera nodes with different view points. We represent a human pose using 13 joints, as shown in Figure 2. The 13 joints include a left shoulder, a right shoulder, a left elbow, a right elbow, a left wrist, a right wrist, a left hip joint, a right hip joint, a left knee, a right knee, a left foot and a right foot.

The Samba motion capture system is a data-driven 3D human pose reconstruction system to improve the accuracy and robustness. A 3D human pose is reconstructed based on input images (*i.e.*, 2D marker positions) and the human pose database, which consists of publicly available human motion data sets and human poses collected using Samba. Each pose in our database is represented as a 39-dimensional pose vector (13 joints × 3 dimensions).

**Figure 3.**
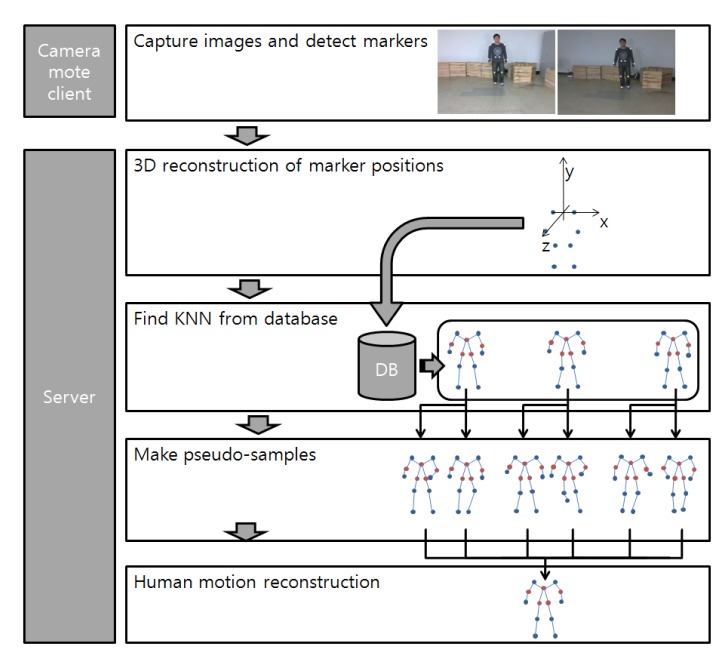
A flowchart of the Samba motion capture system based on a wireless camera sensor network.

The overall computational flow of the Samba motion capture system is given in Figure 3. While a human pose is reconstructed using 13 joint positions, the system uses only eight markers, and the locations of those eight markers are circled in Figure 2. The use of a reduced number of markers makes it more convenient for general users, and we can also reduce the computation time on camera nodes. From an extensive experimental study, we have found that eight is the minimum number of marker locations that can provide the full 3D reconstruction of 13 joints with almost no loss.

The Samba motion capture system operates as follows. Each camera node tracks eight markers in its image frame and transmits the locations of markers to a server at a fixed time intervals. When the server receives marker locations from camera nodes, it computes the 3D positions of markers using multi-view geometry based on the camera network calibration parameters computed during the calibration step (for more detail, see [2]). The computed 3D markers are aligned to be compared with 3D poses in the human pose database. From the human pose database, *K* poses, which are close to the computed 3D marker locations, are selected. We then generate pseudo-poses from the *K* selected poses to form a local pose space. We first reduce the dimensionality of the local pose space using principal component analysis (PCA) to reduce the computation time and noise. We then optimize the corresponding human pose in the lower dimension using constraints on human motion and the human joint model. The optimized human pose is reprojected to the original pose space to obtain a 3D human pose. In the following section, we describe each step of Samba in detail.

## 4. Data-Driven Human Pose Estimation

Samba implements the data-driven human pose estimation method from [15]. While we can reduce the computation time and improve the 3D reconstruction accuracy using a local pose space constructed from a human pose database based on [15], we have found that the method suffers when data points in the human pose database are clustered or sparse. To address this issue, Samba adds an extra step to generate pseudo-poses around the current pose in order to better represent the local human pose space. In the remainder of this section, we describe the data-driven human pose estimation method [15] and the pseudo-pose generation method designed for Samba.

### 4.1. Local Pose Space Method

Suppose that *i* is the current frame number. From images of at least two different view points, a 3D joint marker vector, *x_i_* ∈ ℝ^24^ (8 markers × 3 dimensions), is computed, and *K* poses $\left\{q_{i}^{\left(k\right)}|1\leq k\leq K\right\}$that are close to the computed marker positions are selected from the database. Note that not all eight markers are required to select *K* neighboring poses. For each pose, $q_{i}^{\left(k\right)}$, we randomly generate *N* pseudo-poses, as described in the next section. Let $Q_{i}=\{q_{i}^{\left(k,n\right)}|1\leq k\leq K,1\leq n\leq N\}$ be a set of *KN* pseudo-poses. Pose vectors in *Q_i_* form a local pose space for the current pose.

Let *p_i_* ∈ ℝ^39^ (13 marker positions × 3 dimensions) be the mean of *Q_i_*. PCA is applied to the covariance matrix, Λ*_i_*, of *Q_i_* to obtain an estimate of the current pose vector, *q̄_i_* ∈ ℝ^39^, as follows:

(1) $${\bar{q}}_{i}=p_{i}+U_{i}w_{i}$$

where *w_i_* ∈ ℝ*^n^* is a low-dimensional representation of *q̄_i_* and *U_i_* ∈ ℝ^39×^*^n^* is a projection matrix. The vector, *w_i_*, is constructed from the *n* largest eigenvalues of the covariance matrix, Λ*_i_*, and the columns of *U_i_* are the corresponding eigenvectors.

In order to recover the low-dimensional current pose, we solve the following energy minimization problem.

(2) $$w_{i}^{*}=\arg\underset{w_{i}}{\min}E_{p}(w_{i})+\alpha E_{e}(w_{i})+\beta E_{s}(w_{i})$$

where *E_p_*, *E_e_* and *E_s_* are energy terms and *α* and *β* are weighting parameters. *E_p_* measures the deviation from the distribution of local poses; *E_e_* measures the reconstruction error, and *E_s_* measures the smoothness of motion. These three energy terms are described below.

**The prior term** (*E_p_*): We assume that a pose in the local pose space is distributed according to a multivariate Gaussian distribution as follows:

$$P({\bar{q}}_{i}|Q_{i})=\frac{1}{{\left(2\pi \right)}^{\frac{d}{2}}{\left|\Lambda_{i}\right|}^{\frac{1}{2}}}\exp\left(-\frac{1}{2}{\left({\bar{q}}_{i}-p_{i}\right)}^{T}{\Lambda_{i}}^{-1}({\bar{q}}_{i}-p_{i})\right)$$

where *d* = 39, the dimension of *q̄_i_*, and *p_i_* and Λ*_i_* are the mean and covariance matrix of pseudo-poses in *Q_i_*. We desire to find *q̄_i_*, which is consistent with *KN* pseudo samples; hence, *q̄_i_* maximizes the density function. Maximizing the density function is equivalent to minimizing the following energy function.

(3) $$\begin{array}{ll} E_{p}(w_{i}) & ={\left({\bar{q}}_{i}-p_{i}\right)}^{T}\Lambda_{i}^{-1}({\bar{q}}_{i}-p_{i})\\ & ={\left(U_{i}w_{i}\right)}^{T}\Lambda_{i}^{-1}(U_{i}w_{i}) \end{array}$$

**The reconstruction error term** (*E_e_*): The reconstruction error term measures the distance between detected marker locations and marker locations from the estimated pose.

(4) $$E_{m}(w_{i})=\left\|x_{i}-f({\bar{q}}_{i})\right\|$$

where *f*(*q*) is a function that extracts eight marker positions from the pose, *q*.

**The smoothness term** (*E_s_*): *E_s_* measures the smoothness of the estimated pose from two previous estimated poses, *q̄_i_*_−1_ and *q̄_i_*_−2_, to make the current pose consistent with previous estimates and defined as follows:

(5) $$E_{s}(w_{i})=\left\|{\bar{q}}_{i}-2{\bar{q}}_{i-1}+{\bar{q}}_{i-2}\right\|$$

Combining all the energy terms from Equations (3)–(5) and substituting *q̄_i_* using Equation (1), the energy minimization problem (2) becomes:

(6) $$\begin{array}{ll} w_{i}^{*} & =\arg\underset{w_{i}}{\min}{\left(U_{i}w_{i}\right)}^{T}{\Lambda_{i}}^{-1}(U_{i}w_{i})\\ & +\alpha \left\|x_{i}-f(p_{i}+U_{i}w_{i})\right\|\\ & +\beta \left\|p_{i}+U_{i}w_{i}-2{\bar{q}}_{i-1}+{\bar{q}}_{i-2}\right\| \end{array}$$

The above optimization problem is solved for $w_{i}^{*}$ using the Levenberg–Marquardt method [17], and the current pose, *q̄_i_*, is computed using Equation (1).

### 4.2. Pseudo-Pose Generation

The success of the local pose space method depends on how well a local pose space is represented by data points from the database. Since it is not practical to collect every possible human pose and store them in a database, we propose the use of pseudo-poses to better represent local pose spaces. Each pseudo-pose is generated randomly using an existing pose from the database, while maintaining human motion and joint constraints.

A pseudo-pose is generated by adding a little noise to a joint angle. Since each entry in our human pose database is a joint position vector representing positions of 13 joints in 3D, in order to generate a pseudo-pose, we need to compute joint angles from the joint position vector. Since it is faster to use joint position vectors when estimating the current pose vector, and we do not need to compute all joint angles, a joint position vector is a more convenient representation for our human pose database.

There are two types of joints: joints with one degree of freedom (DOF) and joints with two degrees of freedom (see Figure 2). In a general human body model, shoulders, hip joints and the back bone have three degrees of freedom. However, we do not consider the twist along the direction of the bone for joints with three DOFs, since such twisting causes a large variation. Hence, we treat the joints with three DOFs as joints with two DOFs when generating pseudo-poses. In what follows, we describe how a pseudo-pose is generated for each type of joint.

#### 4.2.1. One-DOF Joints

**Figure 4.**
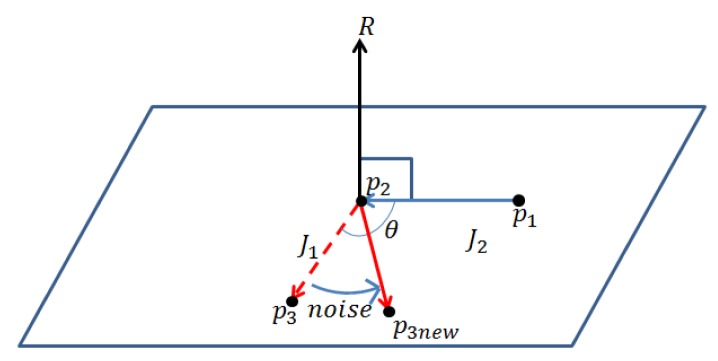
Generation of a new joint angle for a one-degree of freedom (DOF) joint. The joint, *p*_3_, is moved to *p*_3_*_new_* by adding a noise to the joint angle, ∠*p*_1_*p*_2_*p*_3_. The axis of rotation is *R*.

Let us consider a joint with one DOF formed by joints *p*_1_, *p*_2_ and *p*_3_ (see Figure 4). The corresponding joint angle is the angle between two lines, $\vec{p_{1}p_{2}}$ and $\vec{p_{2}p_{3}}$. Let $J_{1}=\vec{p_{2}p_{3}}$ and $J_{2}=\vec{p_{1}p_{2}}$. Then, the angle, ∠*p*_1_*p*_2_*p*_3_, can be computed as follows:

(7) $$\theta =\pi -\arccos\left(\frac{J_{1}^{T}J_{2}}{\left\|J_{1}\right\|\left\|J_{2}\right\|}\right)$$

The rotation axis, *R*, is:

(8) $$R=\frac{J_{1}\times J_{2}}{\left\|J_{1}\right\|\left\|J_{2}\right\|}$$

where × is a cross product between two vectors. By rotating *p*_3_ by a small noise with respect to *R*, we assign a new joint angle, ∠*p*_1_*p*_2_*p*_3_, to the joint, *p*_2_, and obtain *p*_3_*_new_*.

#### 4.2.2. Two-DOF Joints

Let us consider a joint with two DOFs. *J*_1_, *J*_2_, *p*_1_, *p*_2_ and *p*_3_ are defined the same as in Section 4.2.1. We add a small noise to each degree of freedom and obtain a new joint angle for the two-DOF joint.

**Figure 5.**
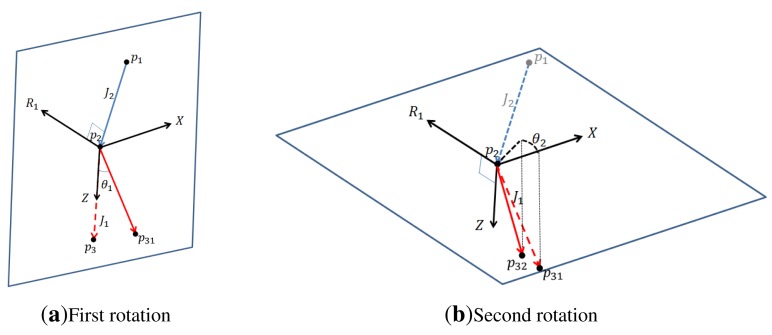
Generation of a new joint angle for a two-DOF joint. The joint, *p*_3_, is first moved to *p*_31_ by adding a noise to the joint angle, ∠*p*_1_*p*_2_*p*_3_, with respect to *R*_1_ (**a**) and then moved to *p*_32_ by rotating with respect to *Z* (**b**).

Let *Z* be a unit vector in the direction of $\vec{p_{2}p_{3}}$ (see Figure 5). Let *υ* be a vector from the right shoulder to the left shoulder. We obtain the *x*-axis of the local coordinate for *J*_1_ based on *υ* by the following equation.

(9) $$X=\frac{\upsilon -(\upsilon^{T}Z)Z}{{\left\|\upsilon -(\upsilon^{T}Z)Z\right\|}_{2}}$$

We also obtain the axis of rotation, *R*_1_, by *R*_1_ = *Z* × *X*.

For simplicity, let us assume that *X* = (1, 0, 0), *R*_1_ = (0, 1, 0), and *Z* = (0, 0, 1). Then, *p*_3_ = (0, 0, *L*), where *L* is the length of $\vec{p_{2}p_{3}}$. We now rotate *p*_3_ with respect to *R*_1_ by *θ*_1_ using the following equation. The resulting new position is *p*_31_.

(10) $$p_{31}=\left[\begin{array}{ccc} \cos\theta_{1} & 0 & \sin\theta_{1}\\ 0 & 1 & 0\\ -\sin\theta_{1} & 0 & \cos\theta_{1} \end{array}\right]p_{3}$$

Next, we rotate *p*_31_ by *θ*_2_ with respect to *Z* using the following equation to obtain *p*_32_.

(11) $$p_{32}=\left[\begin{array}{ccc} \cos\theta_{2} & -\sin\theta_{2} & 0\\ \sin\theta_{2} & \cos\theta_{2} & 0\\ 0 & 0 & 1 \end{array}\right]p_{31}$$

For a pose, *q*, from the human pose database, we generate *N* pseudo-poses by adding a small noise to each joint angle, as described above. By adding noises to the existing pose, we obtain a set of pose variations, which is useful for representing the local pose space. In experiments, we have found that *N* = 10 gives the best tradeoff between the reconstruction error and the computation time.

### 4.3. Initial Values for Optimization

For the faster convergence of the nonlinear optimization problem given in Equation (6), we initialize *w_i_* using a linear least squares solution of the following problem. Given $Q_{i}=\{q_{i}^{\left(k,n\right)}\}$, a set of pseudo-samples for the *i*-th pose, let $r_{i}^{\left(k,n\right)}=f\;\left(q_{i}^{\left(k,n\right)}\right)$ for 1 ≤ *k* ≤ *K* and 1 ≤ *n* ≤ *N*, where *f*(*q*) is a function that extracts eight marker positions from the pose, *q*. We concatenate $r_{i}^{\left(k,n\right)}$ and construct a matrix $R_{i}=\left[r_{i}^{\left(1,1\right)},r_{i}^{\left(1,2\right)},\ldots ,r_{i}^{\left(K,N\right)}\right]$, such that *R_i_* is a 24 × *KN* matrix of eight marker positions of pseudo-poses. Let *γ_i_* ∈ ℝ*^KN^* be a weight vector. We want to find *γ_i_*, which minimizes ‖*x_i_* − *R_i_γ_i_*‖^2^, where *x_i_* is the observed pose vector. The linear least squares solution $\gamma_{i}^{*}={\left(R_{i}^{T}R_{i}\right)}^{-1}R_{i}^{T}x_{i}$ minimizes the error of representing *x_i_* using a linear combination of pseudo-poses. Given $\gamma_{i}^{*}$, the initial pose is computed as ${\bar{q}}_{i}=R_{i}\gamma_{i}^{*}$. We then project *q̄_i_* on to the low-dimensional space to obtain the initial value for *w_i_*, which is used in the nonlinear optimization routine.

## 5. System Implementation

Our implementation of the Samba motion capture system consists of a server and a wireless camera sensor network with two camera nodes. While a larger number of camera nodes is possible, we used the minimum possible number of camera nodes, which is two, to show the feasibility of Samba. Each camera node is equipped with a Gumstix Overo Fire computer-on-module [18] and a Logitech C250 webcam. A camera node runs a client program written using the OpenCV library, which detects and tracks markers and transmits marker positions to the Samba server regularly.

### 5.1. Camera Node

**Figure 6.**
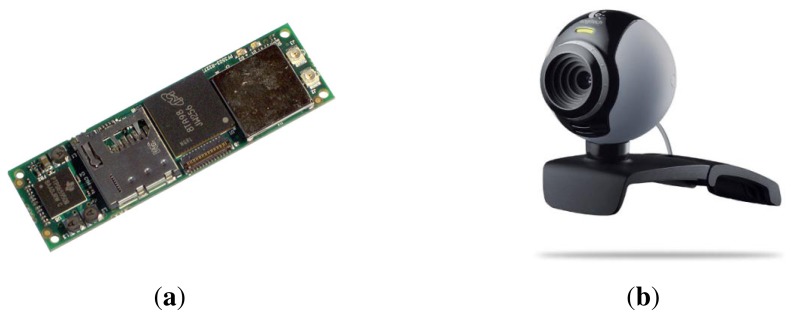
(**a**) Gumstix Overo Fire computer-on-module. (**b**) Logitech C250 webcam.

A combination of a Gumstix Overo Fire computer-on-module and a webcam is used as a camera node (see Figure 6). Gumstix Overo Fire has an ARM Cortex-A8 processor, which can be run up to 720MHz with 512MB of RAM and 512MB of ROM. The module can provide wireless connectivity, such as Bluetooth and Wi-Fi, and it weighs less than 50 g. Compared to other existing camera nodes, such as CITRIC [16], the developed Gumstix-based node has a higher processing power and supports floating-point operations, which is required to run computer vision programs using OpenCV. However, it is still challenging to process high resolution images using Gumstix in real time. Since Gumstix does not support OpenCV 2.x, we have ported OpenCV 1.0. While a Logitech C250 webcam is used for Samba, other cameras with Linux drivers can be used. Wi-Fi is used to communicate between camera nodes and the Samba server.

Table 1 shows image processing times at a camera node for different image sizes. The server program runs at 30 ms per frame; hence, a camera node does not need to run faster than 30 ms per frame. When the image resolution is 640 × 480, it takes 423 ms on average to process one image, while a 320 × 240 image takes 131 ms on average. Hence, we have chosen to use images at 160 × 120 resolution, which takes about 64 ms per frame on average. Due to the processing time at a camera node, the overall frame rate of the Samba motion capture system is 16 frames per second.

### 5.2. Samba Server

The Samba server, which is a notebook computer, receives 2D marker positions from camera nodes. After camera initialization, camera nodes start taking images and report marker positions. The server reconstructs 3D marker positions using camera calibration parameters and performs data-driven human pose estimation using the human pose database, as discussed above.

### 5.3. Human Pose Database

**Figure 7.**
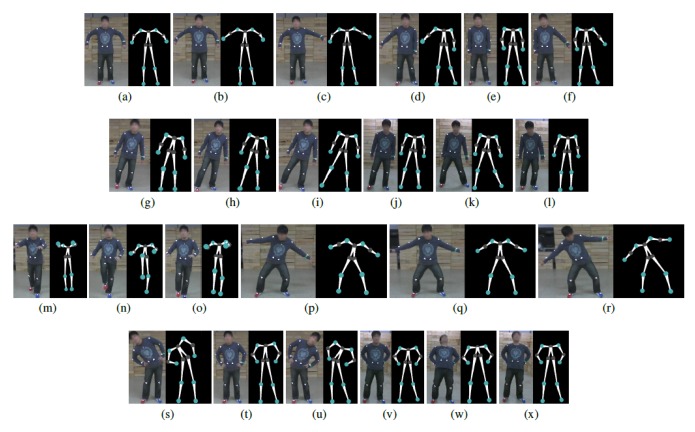
Examples of actions from the local human pose database. (**a**)–(**c**) Arm swing. (**c**)–(**f**) Arm lifting. (**g**)–(**i**) Fake motion (soccer). (**j**)–(**l**) Zig-zag. (**m**)–(**o**) Knee kick. (**p**)–(**r**) Surfing. (**s**)–(**u**) Stretching 1. (**v**)–(**x**) Stretching 2.

The human pose database is constructed from two sources: a local database and the CMU motion capture data [19]. A local database is a collection of 3D human poses collected using Samba. For the collection of 3D human poses, a human subject wears 13 markers at joint positions. A total of eight different actions are collected: two upper body actions, two lower body actions and four whole body actions. See Figure 7 for examples. The CMU motion capture data is converted to align the 13 joint positions and included in the Samba human pose database. For an efficient retrieval of neighboring poses, a k-d tree is used. The incorporation of an existing human pose database, which has been collected using expensive motion capture systems, provides better 3D reconstruction results, while a local database can be used to customize the motion capture system with actions that are not typical in publicly available databases.

### 5.4. Marker Detection and Tracking

Each camera node takes images at a resolution of 160 × 120, due to its computational cost of image acquisition and processing. Moreover, storing images in a Gumstix local memory causes a large overhead for the NAND flash memory. For this reason, images are stored temporarily in the RAM when markers are detected and removed from the RAM before taking the next image.

In Samba, a human subject wears retro-reflective markers, which reflect light when illuminated, since they are easier to detect with a simple intensity-based marker detection algorithm. Given a gray-scale image, the intensity value of each pixel is thresholded. Then, connected component analysis is used to extract blobs. The center of each blob is declared as a marker position.

Samba tracks eight markers, as shown in Figure 2, and they are placed on the following joints: a left shoulder, a right shoulder, a left wrist, a right wrist, a left knee, a right knee, a left foot and a right foot. We assume that a human subject starts with a standard pose, in which all marker positions are known, and all marker positions are initialized based on the standard pose. Then, each marker is tracked using a nearest neighbor filter. Since the frame rate is high for low-resolution images, we can reliably track all markers using nearest neighbor filtering.

### 5.5. 3D Reconstruction of Markers

As the Samba server receives two sets of marker positions from camera nodes, it performs 3D reconstruction of markers using the extrinsic camera parameters found during the calibration step based on the method described in [20]. Since the reconstructed 3D marker positions are aligned with respect to the camera coordinate system, they cannot be directly compared to human pose vectors in the database. We rescale and align 3D marker positions, such that an observed pose is consistent with the coordinate system used in the human pose database.

## 6. Experimental Results

An extensive set of experiments is performed to validate the performance of the Samba motion capture system. A notebook computer is used as a server, which receives data wirelessly from camera nodes. In order to measure reconstruction errors, we have defined the length of the tibia (lower leg) as 45 cm. The reconstruction error is the average 3D position error of 13 joints over each action sequence.

### 6.1. Number of Neighbors (K)

**Figure 8.**
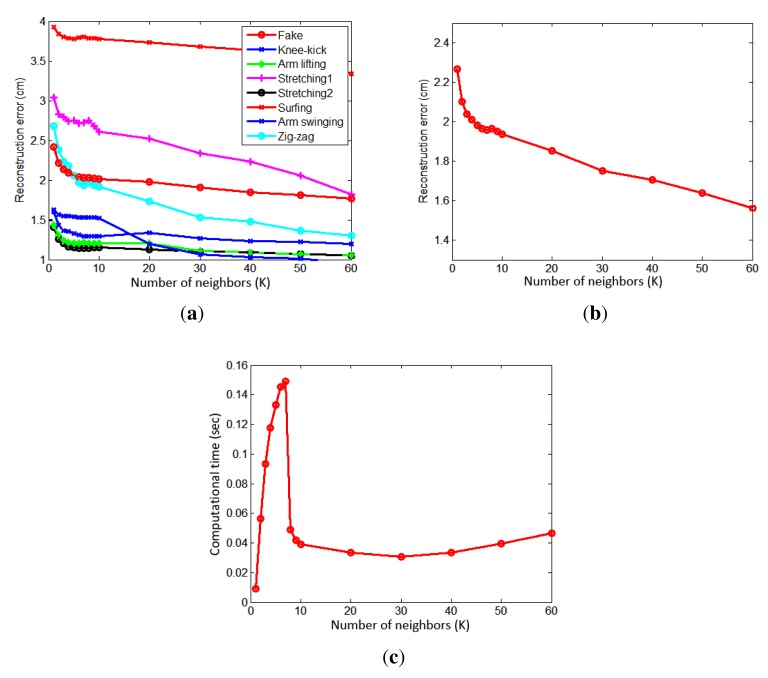
(**a**) Reconstruction errors of eight actions at different numbers of neighbors. (**b**) Average reconstruction errors of all eight actions at different numbers of neighbors. (**c**) Computation times at different numbers of neighbors.

A local pose space is constructed using neighboring poses. In order to determine the effect of the number of neighbors, we varied the number of neighbors, *K*, and measured the reconstruction errors and required computation times. The results are based on image sequences of eight actions, which are not in the database and each action sequence contains about 900 frames. For all eight actions, we computed reconstruction errors at different *K*, and they are shown in Figure 8a. The surfing action gave the worst error, due to a large variation when both arms are swung.

The average reconstruction error of all eight actions as a function of the number of neighbors is shown in Figure 8b. As expected, the reconstruction error decreases as the number of neighbors, *K*, is increased. The computation times for different *K* are shown in Figure 8c. The computation time increases rapidly and decreases when *K* = 8. Then, it slowly decreases, until *K* = 30, and, then, slowly increases again. This is due to the fact that the optimization routine requires more time for finding a solution when the number of data is small (the optimization problem becomes numerically unstable).

### 6.2. Number of Pseudo-Poses (N)

**Figure 9.**
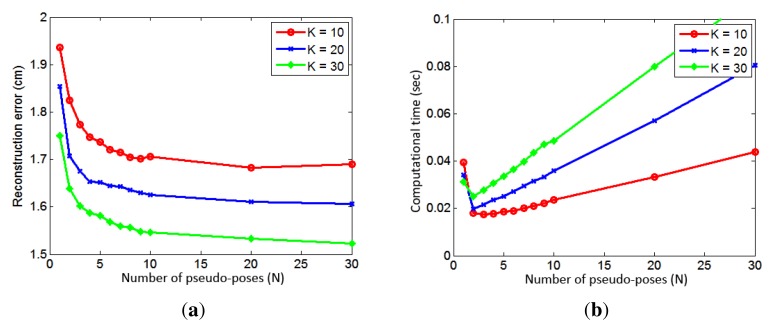
(**a**) Reconstruction errors at different numbers of pseudo poses for *K* = 10, *K* = 20 and *K* = 30. (**b**) Computation times at different numbers of pseudo poses for *K* = 10, *K* = 20 and *K* = 30.

**Figure 10.**
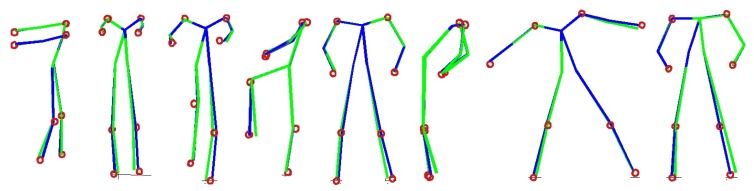
Examples of reconstructed poses. The red dots indicate the ground-truth positions of eight markers. The green lines are reconstruction results without pseudo-poses, and the blue lines are reconstruction results with pseudo-poses.

We have also evaluated the effect of pseudo-poses in terms of the reconstruction error and computation time. Figure 9a shows reconstruction errors as more pseudo-poses are used to represent the local pose space. We have tested three different values of *K* (10, 20 and 30). Again, the results are based on image sequences of eight actions, which are not in the database, and each action sequence contains about 900 frames. For all three cases, the reconstruction error decreases rapidly until *N* = 10 and, then, decreases slowly. Note that, when *N* = 1, no pseudo-pose is used, and the local pose space is constructed using *K* neighboring poses. The result clearly indicates the benefit of using pseudo-poses. The reduction of the reconstruction error from using pseudo-poses is about 14% when *K* = 10 and *N* = 10. Figure 10 shows some examples where the 3D reconstruction of a pose is improved by the use of pseudo-poses. The required computation times at different values of *N* are shown in Figure 9b. We see that the computation time increases linearly with *N* when *N* is larger than two. It is interesting to note that the computation time is significantly reduced when pseudo-poses are used. When *K* = 10 and *N* = 10, the average computation time is 0.0235 sec and the optimization routine requires about 49 iterations on average. However, when *K* = 100 and pseudo-poses are not used, it requires about 0.0886 sec and 78 iterations on average. The use of pseudo-poses has reduced the computation time by 73%. When *K* = 10 and *N* = 1, it still requires about 0.04 sec. This illustrates that pseudo-poses can better represent the local pose space with a dense set of data points, which results in faster convergence. Hence, we can better reconstruct a 3D human pose using pseudo-poses given a limited amount of time.

We have found that the best trade-off between the reconstruction error and computation time is when *K* = 10 and *N* = 10. These values are used for the experiments described below.

### 6.3. CMU Motion Capture Database

**Figure 11.**
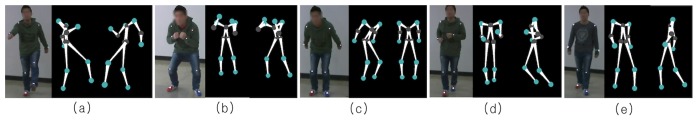
Reconstruction of poses from the CMUmotion capture database. (**a**) March. (**b**) Guard. (**c**) Jump. (**d**) Run. (**e**) Walk.

The human pose database in Samba is a combination of the CMU motion capture data and locally collected motions, as described above. See Figure 7 for examples of locally collected motions. The CMU motion capture data used in the human pose database consists of five motions: march, guard, jump, run and walk. As shown in Figure 11, the Samba motion capture system can reconstruct poses that are from the CMU motion capture database, as well.

### 6.4. Demonstration of the Samba Motion Capture System

**Figure 12.**
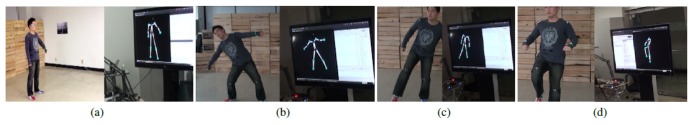
A real-time demonstration of the Samba motion capture system. (**a**) Arm lifting. (**b**) Surfing. (**c**) Fake motion. (**d**) Knee kick.

Some snapshots from a demonstration of the Samba motion capture system are shown in Figure 12. For each photo in Figure 12, the left half of the photo shows a human subject performing a motion, and the right half of the photo shows the reconstructed 3D pose displayed on a monitor, demonstrating the real-time operation of Samba.

**Figure 13.**
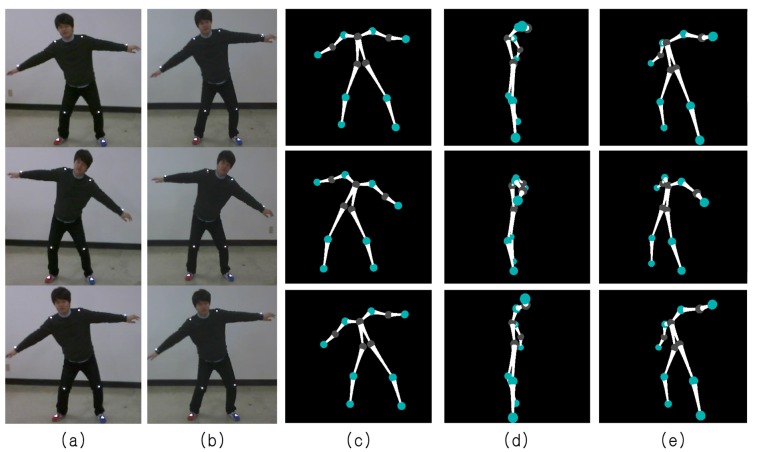
3D reconstruction results from the surf motion by another user. (**a,b**) The images taken by camera nodes. (**c**–**e**) The reconstructed poses from different view points.

Figure 13 shows reconstruction results for a new user. While only eight markers are used by Samba, Samba reconstructs the entire human pose reliably throughout the action sequence. Note that the surfing motion had the worst reconstruction error. However, the reconstruction results are quite impressive.

**Figure 14.**
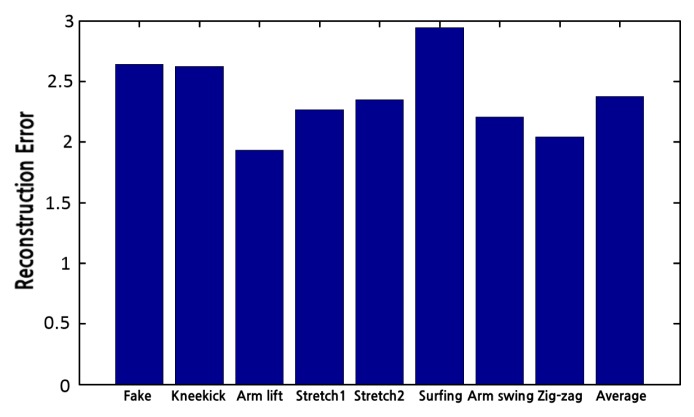
Reconstruction errors of eight actions using the Samba motion capture system. The average reconstruction error is 2.37 cm.

We have performed eight actions from the local pose database in front of the Samba system. The resulting reconstruction errors are shown in Figure 14. The reconstruction errors from the Samba system are slightly larger than the reconstruction errors reported in Section 6.1. This is due to the fact that the local database is constructed using images at 640 × 480 resolution, which results in the better localization of markers. However, the proposed system reconstructs various motions with an average error of 2.37 cm at 16 frames per second, which is small enough to represent a wide range of motions reliably. This demonstrates the feasibility of the Samba system as a low-cost alternative for a real-time motion capture system.

**Figure 15.**
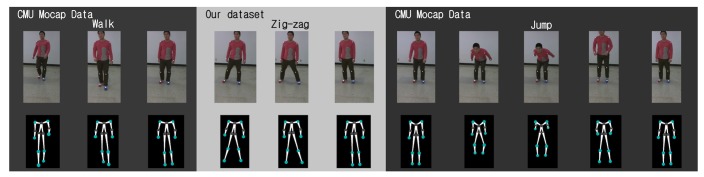
Reconstruction of a composite action: walk (CMU), zigzag (local) and jump (CMU).

**Figure 16.**
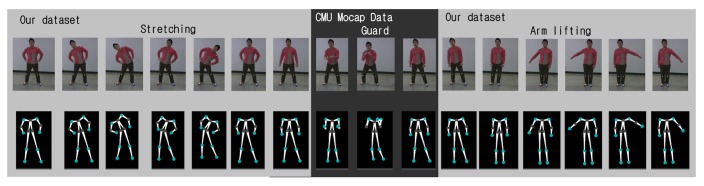
Reconstruction of a composite action: stretching (local), guard (CMU) and arm lifting (local).

One limitation of the data-driven human pose estimation method is that it may not be able to reconstruct a pose that is significantly different from the poses in the database. This problem can be addressed by adding more poses to the database, as allowed by the Samba system. The next issue is whether the system can make a smooth transition from a pose from one database to a pose from another database. We can make a transition between databases by introducing more importance to the smoothness term in Equation (6) and increasing the number of pseudo-poses and noises added to pseudo-poses. Figures 15 and 16 show transitions between different databases, demonstrating that Samba can reliably capture complex composite actions in real time.

### 6.5. A Comparison to Kinect under the Bright Lighting Condition

**Figure 17.**
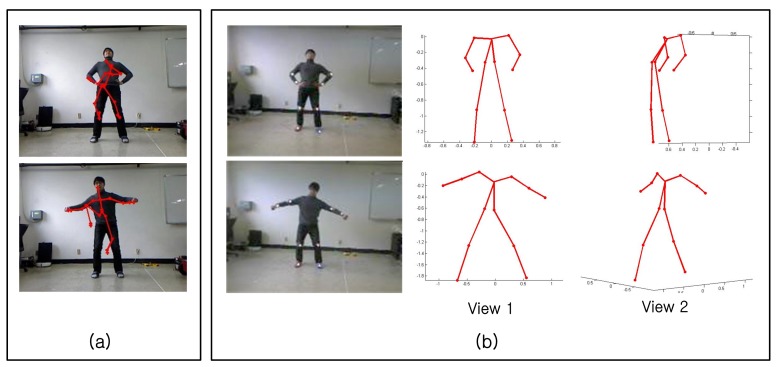
A comparison between Kinect and Samba under the bright lighting condition. (**a**) 3D pose reconstruction using Kinect. (**b**) 3D pose reconstruction using Samba.

As described in the introduction, Kinect does not operate well under the bright lighting condition [3]. In this section, we compare the proposed method and pose estimation by Kinect under the bright lighting condition. The experimental setup is the same as before, but we conducted the experiment on a sunny day with windows open. Figure 17 shows results from Kinect and the proposed method under the same environment. Results for Kinect are obtained using the Image and Data Acquisition toolboxes in MATLAB with the Kinect support. As shown in the figure, Kinect fails to reconstruct the human pose correctly under the bright lighting condition, while Samba successfully reconstructs 3D human poses. Note that the resolution of images taken by Samba is 160 × 120, which is lower than the RGB image taken by the Kinect camera.

## 7. Conclusions

In this paper, we have described the design and implementation of Samba, a real-time motion capture system, which is based on a low-cost portable wireless camera sensor network. A new class of camera nodes is constructed in order to process images in real time. We have also reduced the required number of joint markers to eight to make it easier for general users. The proposed system provides accurate reconstruction of 3D human poses at 16 frames per second using the data-driven pose estimation method augmented with pseudo-poses. An ability to combine existing human pose databases with a locally-generated pose database makes Samba scalable and a practical solution for general motion capture applications.

## Figures and Tables

**Table 1.** Processing times for different image sizes by a camera node.

**Image size**	160 × 120	320 × 240	640 × 480
Average	64 ms	131 ms	423 ms
Maximum	102 ms	360 ms	925 ms
Minimum	62 ms	75 ms	324 ms
